# Clinical analysis of three patients with multiple endometrial cancer lesions treated by hysteroscopic surgery combined with progesterone: Case report

**DOI:** 10.1097/MD.0000000000034329

**Published:** 2023-07-14

**Authors:** Li Wang, Rimin Cong, Lili Zhang, Danni Zhang, Juntong Wu

**Affiliations:** a Department of Obstetrics and Gynecology, The 964th Hospital, Changchun, Jilin Province, China; b Changchun University of Chinese Medicine, Changchun, Jilin Province, China.

**Keywords:** early endometrial cancer, fertility preservation, hysteroscopy, multiple lesions, progesterone

## Abstract

Endometrial cancer (EC) is one of three common malignant tumors of the female reproductive tract, exceeded by cervical cancer. It has an incidence rate ranging from 3% to 10% with an increasing trend in recent years.

**Patient concerns:**

To analyze the clinical effectiveness of hysteroscopic surgery combined with progesterone therapy in patients with multiple lesions of endometrial cancer with fertility preservation.

**Diagnoses:**

Multiple endometrial cancer lesions were present.

**Interventions:**

Three patients presented with menstrual cycle disorders, irregular vaginal bleeding, and endometrial thickening. Diffuse and local bulges of the endometrium can be seen under hysteroscopy. The histopathological results showed varying degrees of complex endometrial hyperplasia with canceration. Progesterone receptor was detected in lesion tissues through immunohistochemical examination.

**Outcomes:**

Case 1 fully recovered within 2 months, case 2 within 3 months, and case 3 within 9 months.

**Lessons:**

Our data suggested the clinical efficacy of hysteroscopic surgery combined with progesterone therapy in patients with early multiple endometrial cancer, providing the precious experience of the clinical presentation.

## 1. Introduction

Endometrial cancer (EC) is 1 of 3 common malignant tumors of the female reproductive tract, exceeded by cervical cancer. It has an incidence rate ranging from 3% to 10% with an increasing trend in recent years.^[1]^ About 65% of EC patients have no reproductive history. Surgery is the first choice for EC treatment, but it can lead to loss of fertility and affect the patient’s quality of life. This paper analyzes the data from 3 cases with early multi-focal EC which were treated by hysteroscopic surgery combined with progesterone in our hospital. Progesterone receptor was detected in lesion tissues, and patients had strong desires for fertility preservation. The clinical features, diagnostic parameters, and treatment measures are analyzed and summarized.

## 2. Case presentation

All patients were eligible for fertility-preserving therapy^[2]^: age ≤ 40 years old with a strong desire to preserve fertility; the lesion biopsy was characterized as well-differentiated endometrioid adenocarcinoma; immunohistochemical examination: estrogen receptor (ER) and progesterone receptor (PR) were positive; serum CA125 was normal; imaging evaluation showed the lesion was limited to the endometrium with myometrial invasion, cervical, and extrauterine lesions; IA clinical stage according to the stage guideline from International Federation of Obstetrics and Gynecology; normal liver and kidney function; no contraindications to drug treatment; all subjects and their legal guardians informed consent was signed by all patients, and they underwent regular follow-ups; all clinical trials were approved by the Ethics Committee of the 964th Hospital.

The effect of this treatment was divided into 4 groups: complete reversal: pathological examination after treatment showed that the patient’s lesions completely disappeared without any cancer or atypical hyperplasia; partial reversal: pathological results showed atypical hyperplasia; no reversal: no response to treatment and the lesion persisted; recurrence: cancer tissue reappeared in patients after reversal.

### 2.1. Case 1

**Figure 1.**
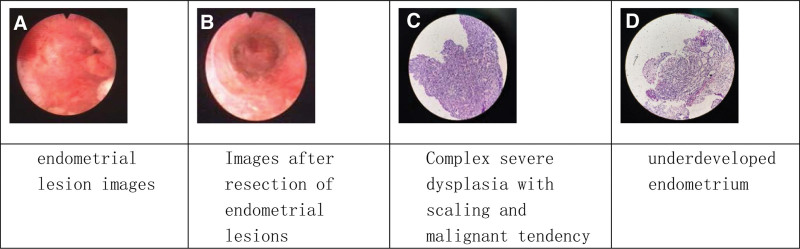
Hysteroscopy and pathological examination of patient 1 before and after treatment.

A 35-year-old female with menarche at the age of 17, no normal menstrual cycle, intermittent vaginal bleeding, BMI: 27.3. A diagnostic curettage was performed in 2008 due to irregular vaginal bleeding. No treatment was performed as the endometrial pathology showed simple hyperplasia with local complex hyperplasia in endometrium. From 2009 to 2012, three ovulation induction treatments were performed. In 2012, due to ectopic pregnancy, laparoscopic left fallopian tube incision and embryo extraction were performed. In the first visit to our hospital in April 2014, the patient received hysteroscopy and surgery. The depth of the uterine cavity was about 9.5 cm, showing multiple polypoid lesions. It was found that the endometrial hyperplasia lesions exceeded half of the depth of the uterine cavity, showing diffuse and local bulges in 5 spots. The patient had a strong desire to preserve the reproductive function and the uterus, and received hysteroscopic surgery to remove the lesion. The depth of resection was 2 to 3 mm below the lesion, and the surrounding area of the lesion was cut to 2 to 3 mm of normal tissue. Histopathological results of resection: the endometrium was highly complex atypical hyperplasia with squamous change and malignant transformation; immunohistochemical results: PR 30%. The patient received postoperative oral progesterone therapy. The treatment methods, process, and outcomes are shown in Table 1. Endometrial reversal occurred within 2 to 3 months of treatment either in endometrial atypical hyperplasia or developing EC. Recurrence occurred within 3 to 11 months after drug withdrawal. After receiving the same treatment for 2 to 3 months, the patient showed reversal. The endometrium showed decidua or polypoid hyperplasia, and poor development. Now the patient has stopped the drug for 4 years and has been amenorrheic for 2 years with a levonorgestrel-releasing intrauterine system (LNG-IUS) placed in the uterine cavity. No recurrence was found at 2 to 3 months follow-up. Monitoring indicators included hysteroscopy, endometrial histopathology, ultrasound monitoring of endometrial thickness, pelvic MRI to examine pelvic lymph nodes, as well as liver function, kidney function, and tumor markers (Fig. 1).

**Table 1:** Patient 1 treatment process and outcomes (8 years)

Time line (months)	Hysteroscopy examination	Pathological results	Treatment	Outcomes
0	Examination and diagnosis	Complex severe dysplasia with scaling and malignant transformation (Fig. 1C)	Medroxyprogesterone acetate 500 mg, qd, changed to 250 mg, qd after 1 month due to serious side effects	
2	Endometrial biopsy	Partial decidua	Medroxyprogesterone acetate 250 mg, qod, for 3 months	Complete reversal
6	Endometrial biopsy	Simple endometrial hyperplasia with focal complex mild dysplasia endometrial polypoid changes	Medroxyprogesterone acetate 250 mg, qd, for 3 months	Partial recurrence
9	Endometrial biopsy	Endometrial polypoid changes	Medroxyprogesterone acetate 250 mg, qod, for 3 months	Complete reversal
12	Endometrial biopsy	Simple endometrial hyperplasia	Withdrew drug and tried to conceive (without following the doctor’s advice)	No fertilization
23	Endometrial biopsy	Moderately differentiated endometrioid adenocarcinoma with squamous differentiation	Medroxyprogesterone acetate 250 mg, qd, for 3 months	Recurrence and deterioration
26	Endometrial biopsy	Endometrial polypoid hyperplasia without malignancy	Medroxyprogesterone acetate 250 mg, qd, for three months	Complete reversal
28	Endometrial biopsy	Endometrial polypoid hyperplasia without malignancy	Medroxyprogesterone acetate 250 mg, qd, 1 month, 3 months drug withdrawal	No recurrence
32	Endometrial biopsy	Mostly proliferative endometrium, local high-grade neoplasia, malignant tumors not seen	Medroxyprogesterone acetate 250 mg, qd, for 3 months	Recurrence
35	Endometrial biopsy	Endometrial hypoplasia, no abnormalities (Fig. 1D)	Drug withdrawal in order to enter IVF stage	Complete reversal
39	Endometrial biopsy	Small amount of decidual smooth muscle tissue	Intrauterine placement of levonorgestrel IUD (LNG-IUS)	
40–88	Assisted reproduction treatment		Egg retrieval for three times, 2 eggs were retrieved, no fertilization	Not transplanted
89–now	Regular follow-up inspection		After 4 years of drug withdrawal, LNG-IUS was implanted in the uterine cavity	Amenorrhea, no recurrence

IVF = in vitro fertilization, IUD = intrauterine device, LNG-IUS = levonorgestrel-releasing intrauterine system.

### 2.2. Case 2

**Figure 2.**
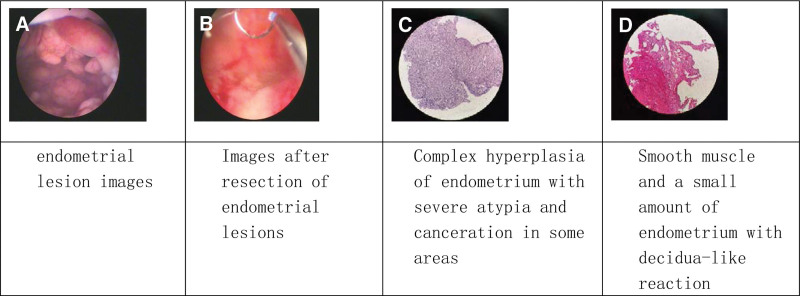
Hysteroscopy and pathological examination of patient 2 before and after treatment.

A 36-year-old female, BMI 31.2, had a full-term cesarean delivery of a boy in 2003 and desired to have a second child. Multiple ultrasound examinations in another hospital showed endometrial thickening. She started treatment in our hospital beginning June 2021. Hysteroscopy result: the depth of the uterine cavity was 10.5 cm, with multiple papillary hyperplasia in the endometrium, and visible abnormal blood vessels on the surface. The lesions exceeded half of the uterine cavity in over 5 spots. Hysteroscopic lesion resection was performed. The depth and extent of resection were the same as in case 1. The pathology results showed complex hyperplasia of the endometrium with severe atypia, cancerous in some areas. Immunohistochemical results: ER++60%, PR++70%. The pathology results after consultation with other hospitals: complex atypical hyperplasia of endometrium with squamous metaplasia, localized cancers. MRI indicated EC (stage IA). The patient had a strong desire to preserve her fertility, so signed a consent form for conservative treatment, and was given systemic and local progesterone therapy after surgery. The medication methods, process, and outcomes were shown in Table 2. Follow-up procedure and time are the same as case 1. Now the patient has stopped taking oral medicine for 9 months. After intrauterine device containing progesterone medicine was removed for 3 months, no recurrence was observed and the process of assisted reproduction started (Fig. 2).

**Table 2:** Patient 2 treatment process and outcomes (over 2 years)

Time line (months)	Hysteroscopy examination	Pathological results	Treatment	Outcomes
0	Examination and diagnosis	Complex hyperplasia of endometrium with severe atypia and cancers in some areas (Fig. 2C). MRI: endometrial cancer (stage IA) is very likely	Medroxyprogesterone acetate 250 mg, bid. Placement of LNG-IUS after surgery	
2	Endometrial biopsy	Decidual degeneration seen in the middle and well-differentiated endometrioid cancer tissue residues in uterine anterior and posterior wall, 10% and 5%, respectively, decidual degeneration on left and right wall, no tumor observed	Medroxyprogesterone acetate 250 mg, qd	Partial reversal
3	Endometrial biopsy	No tumor observed in uterine anterior and posterior wall smooth muscle and a small amount of endometrium, MRI results: the endometrium is slightly thickened and enhanced locally; the anterior wall of the uterus is locally thinned	Medroxyprogesterone acetate 250mg, qod	Complete reversal
6	Endometrial biopsy	Endometrium with interstitial decidua-like changes, no malignancy	Medroxyprogesterone acetate 250 mg, qod, 2 months, then drug withdrawal	No recurrence
12	Endometrial biopsy	Smooth muscle and a small amount of endometrium showed decidua-like reaction (Fig. 2D)		
15–now	Regular follow-up inspection		Drug withdrawal for 9 months, assisted reproduction	Fertility treatment

LNG-IUS = levonorgestrel-releasing intrauterine system.

### 2.3. Case 3

**Figure 3.**
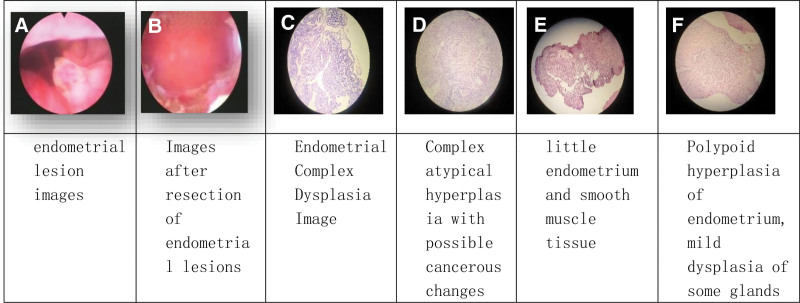
Hysteroscopy and pathological examination of patient 3 before and after treatment.

A 31-year-old unmarried female, no sexual history, irregular menstrual cycle of 30 to 90 days, unstable menstrual flow, occasional dysmenorrhea, BMI 22.7. In 2020, she went to another hospital for the treatment of irregular menstruation. Ultrasound examination showed 2.5 cm endometrial thickness. After oral dydrogesterone treatment for 1 month, drospirenone and ethinyl estradiol tablets for 4 months, ultrasound indicated that the endometrium was 1.7 cm. In October 2021, she visited our hospital for the first time. The results of hysteroscopy showed that the depth of the uterine cavity was about 8.5 cm, multiple papillary polypoid changes, and abnormal blood vessels on the surface. The lesions exceeded half of the uterine cavity in over 5 spots. The patient underwent hysteroscopic endometrial lesion resection with hymen protection. The depth and scope of resection were the same as in case 1. Histopathological results of resection: atypical adenomyomatous endometrial polyps with complex dysplasia. Histochemical results of immunization: PR++90%, ER+++100%, same as the pathological results from other hospital. Systemic and local treatment with progesterone was given after operation. The medication methods, process, and outcomes are shown in Table 3. Follow-up procedure and time are the same as case 1 (Fig. 3).

**Table 3:** Patient 3 treatment process and outcomes (over 1 year)

Time line (months)	Hysteroscopy examination	Pathological results	Treatment	Outcomes
0	Examination and diagnosis	Atypical adenomyomatous endometrial polyp with focal complex dysplasia of the endometrium (Fig. 3C)	Medroxyprogesterone acetate 250 mg, bid	
3	Endometrial biopsy	Complex atypical hyperplasia of endometrium, possible cancerous changes (Fig. 3D)	Medroxyprogesterone acetate 250 mg, bid	Partial reversal
9	Endometrial biopsy	Little endometrium and smooth muscle tissue (Fig. 3E)	Medroxyprogesterone acetate 250 mg, qod	Complete reversal
12	Endometrial biopsy	Polypoid hyperplasia of endometrium, mild dysplasia of some glands (Fig. 3F)	Medroxyprogesterone acetate 250 mg, qd	Recurrence tendency
13–now	Regular follow-up inspection			

## 3. Discussion

### 3.1. Advantages of hysteroscopy-assisted drug therapy

In patients with conservative treatment of EC, the prognosis is better if the tumor cells are well differentiated and the positive rate of ER and PR is high. Previously, conservative treatment was limited to old patients or those with complications who were unable to undergo surgery. With the onset of the trend of EC in younger females, up to 14% of patients were women at reproductive age.^[3]^ It is believed that young stage IA EC patients who desires to keep reproductive function can be treated conservatively, mainly including hysteroscopic resection of lesions of the endometrium, progesterone, gonadotropin-releasing hormone agonist, and other feasible short-term treatments which are alternatives to standard surgery.

Previous conservative method for treatment of EC was mostly repeated endometrial curettage. According to research reports, even experienced gynecologists can only scrape 75% to 80% of the uterine cavity area. Hysteroscopy after dilatation and curettage can still detect about 20% of ridge-shaped endometrial residues. The advantages of hysteroscopy are to remove suspicious lesions under direct vision, reduce the damage to normal endometrium and the muscle layer, and allow the doctor to directly view and remove all possible lesions, especially those near the ostium of the fallopian tube and the internal os of the cervix. Moreover, with hysteroscopy one can observe whether intrauterine lesions recur under direct vision, and take localized biopsy to detect signs of recurrence in time and reduce the chance of intrauterine adhesions.^[4]^

It is reported that hysteroscopy-assisted progesterone therapy as the conservative treatment of EC is limited to patients of well-differentiated and single lesions and 50% of PRs.^[5]^ Progestogens are often used in the conservative treatment of EC. LNG-IUS is a “T”-shaped intrauterine device, which releases levonorgestrel at a constant dose of 20 μg/d after being inserted into the uterine cavity.^[6]^ It can directly act on the endometrium, inhibit the growth of cancer cells, and promote cancer cell apoptosis. According to reports, hysteroscopy combined with drug therapy can achieve a complete remission rate of 97% and a pregnancy rate of 45% in fertility-preserving patients with EC.^[7]^ Through the experimental treatment of three patients, we explored the possibility of hysteroscopic surgery combined with progesterone therapy to preserve fertility in patients with multiple lesions including PR < 50% (case 1).

### 3.2. To explore the feasibility of conservative treatment for multiple lesions of endometrial carcinoma

It has been reported in the literature that the classification of single lesions and multiple lesions in EC patients with fertility requirements is not accurate. No large-scale comparative studies have been performed so far.^[8]^ In this paper, three patients all had multiple lesions in more than 3 spots under the microscope. All patients underwent the resection of lesion tissues and suspicious areas under hysteroscopy. In addition, they were given oral progesterone and local LNG-IUS treatment after the pathological results were reported. Patients were reexamined every month by color Doppler ultrasound. Hysteroscopy was performed if the thickness of the endometrium was more than 5 mm every 3 months. Hysteroscopy and endometrial pathology were routinely performed. The first and second patients achieved complete reversal in 2 and 3 months respectively, while the third patient was at 9 months. The first patient relapsed 3 to 11 months after drug withdrawal. Hysteroscopic lesion resection and the same dose of megestrol acetate were performed again, and the symptoms were completely reversed 2 months later. Three patients were completely reversed after lesion resection under hysteroscopy and high-efficiency medroxyprogesterone acetate treatment. Because all the lesions were removed under hysteroscopy, the effective concentration of each part of the uterus is relatively consistent after systemic drug treatment. LNG-IUS releases drugs in the entire uterine cavity to achieve the local treatment effect of a single lesion. According to the literature, the treatment of endometrial dysplasia to preserve fertility was not limited to single endometrial lesion,^[9]^ which provides the support for the feasibility of the conservative treatment in these 3 cases with multiple lesions. However, due to the relatively small sample size of this study, more subjects are needed to observe the results of hysteroscopic surgery combined with progestin therapy in patients with early multiple EC.

### 3.3. Risk of hysteroscopy-assisted drug therapy

Hysteroscopic resection combined with drug therapy for EC still has a high likelihood for fertility preservation. Many scholars have reported that the recurrence rate was as high as 7% to 10%.^[10]^ Moreover, some believe that hysteroscopic surgery is often accompanied by adhesions in the uterine cavity, which affects endometrial receptivity and brings difficulties to the implantation of fertilized eggs.^[11]^ This is still a problem to be solved. Some articles reported that the fertility-preserving treatment of EC patients had a risk of 4% to 25% combined with malignant ovarian tumors.^[12]^ In recent years, studies with a large sample size had found that ovarian cancer risk is <1% in patients with lesions confined to the uterus.^[13]^ The application of a large amount of progesterone to preserve fertility in patients with EC increased the risk of thrombosis and breast tumors.^[14]^ All three patients were given oral low-dose aspirin to prevent thrombosis, and no thrombosis occurred in any case. During the operation of hysteroscopy, uterine distension media and pressure are required. There are different reports regarding the possibility of tumor cells passing through the fallopian tube to the abdominal cavity which increases the probability of peritoneal metastasis and spread of EC.^[15]^ The reports on above risk occurrence are all limited to the conservative treatment of a single EC lesion. Multiple lesions of EC are more serious than a single lesion. The area of resection with multiple lesions is increased during surgery accompanied with the prolonged operation time. With the increases of tumor cell number and drug dose, whether the risk of recurrence, metastasis, adhesion, ovarian cancer, thrombosis, and other complications of conservative treatment in these patients will be increased deserves our attention and discussion.

## 4. Conclusion

We explored the feasibility of hysteroscopic surgery combined with progesterone treatment in patients with multiple lesions of early EC who have a strong desire to have children under the premise of strict follow-ups and other conditions of conservative treatment. The multiple lesions of EC in these 3 patients were completely reversed after treatment. It is reported that conservative treatment should be limited to patients with a single lesion. Is it possible to expand the treatment to multiple lesions patients? What are the potential complications during treatment? These questions need further in-depth research and discussion after studies with a large sample size.

## Author contributions

**Conceptualization:** Li Wang.

**Data curation:** Lili Zhang.

**Investigation:** Danni Zhang, Juntong Wu.

**Writing – original draft:** Li Wang, Rimin Cong.

**Writing – review & editing:** Li Wang, Rimin Cong, Lili Zhang.
